# Development and analytical performance of an automated screening method for cannabinoids on the Dimension clinical chemistry system

**DOI:** 10.1155/S1463924697000187

**Published:** 1997

**Authors:** D. M. Obzansky, E. G. Gorman, S. P. Kramer, I. S. Masulli, E. A. Nuzzaci, W. F. Skogen

**Affiliations:** Dade Chemistry Systems Inc. PO Box 6101 Newark DE 19714 USA

## Abstract

A fully automated, random access method for the determination of cannabinoids (UTHC) was developed for the Dimension AR and XL clinical chemistry systems. The method utilizes Abuscreen ONLINE reagents and a multianalyte liquid calibrator containing 11-nor-Δ^9^-THC-9-carboxylic acid. Within-run and total reproducibility, determined using NCCLS protocol EP5- T2, was less than 0.6% and 1.6% CV, respectively, at all concentrations. Calibration stability was retained for at least 30 days. An extensive evaluation of non-structurally related drugs and various physiological substances indicated lack of interference in the method. No sample carry-over was observed following a specimen containing 1886 ng/ml 11-nor-Δ^9^-THC-9-carboxylic acid. A 99.1% agreement (N = 445 samples) was found between an EMIT based method on the aca discrete clinical analyser and the Dimension UTHC method.

Dimension clinical chemistry system and aca discrete clinical analyser are registered trademarks of Dade International.


Journal of Automatic Chemistry, Vol. 19, No. 5 (September-October 1997) pp. 169-173

### Development and analytical performance of an automated screening method for cannabinoids on the Dimension® clinical chemistry system

**D. M. Obzansky, E. G. Gorman, S. P. Kramer, I. S. Masulli, E. A. Nuzzaci and W. F. Skogen**
*Dade Chemistry Systems Inc., PO Box 6101, Newark, DE 19714, USA*

*A fully automated, random access method for the determination of cannabinoids (UTHC) was developed for the Dimension® AR and XL clinical chemistry systems. The method utilizes Abuscreen ONLINE® reagents and a multianalyte liquid calibrator containing* 11*-nor-*$\Delta^9$*-THC-*9*-carboxylic acid. Within-run and total reproducibility, determined using NCCLS protocol EP5-T2, was less than* 0·6% *and* 1·6% *CV, respectively, at all concentrations. Calibration stability was retained for at least* 30 *days. An extensive evaluation of non-structurally related drugs and various physiological substances indicated lack of interference in the method. No sample carry-over was observed following a specimen containing* 1886 *ng/ml* 11*-nor-*$\Delta^9$*-THC-*9*-carboxylic acid. A* 99·1% *agreement* (N = 445 *samples) was found between an EMIT® based method on the aca® discrete clinical analyser and the Dimension® UTHC method.*

*Dimension® clinical chemistry system and aca® discrete clinical analyser are registered trademarks of Dade International.*

### Introduction

Laboratory support has been recognized for many years to be an integral part of the hospital service for patients with drug abuse problems. There continues to be laboratory demand for high-quality screening immunoassays for all of the abused drugs including cannabinoids. To provide workstation consolidation and rapid turn-around-times, assays for the detection of cannabinoids need to be available on a high throughput analyser as part of an overall drug of abuse panel. This paper describes the development and performance of a cannabinoid method (UTHC) for the Dimension® AR and XL clinical chemistry systems. The method provides a high quality, rapid screening approach for measuring cannabinoids on these general chemistry analysers. Stable liquid reagents, calibrators and controls are utilized to avoid reagent preparation steps. Since the Dimension® clinical chemistry system is used for the measurement of many other analytes [1], the benefits of workstation consolidation are realized.

### Materials and methods

### *Reagents*

The Dimension® UTHC method utilizes Abuscreen ONLINE reagents (ONLINE is a registered trademark of Roche Diagnostic Systems, Inc., Branchburg, NJ 08876-1760) packaged in Flex™ cartridges (Cat. No. DF95, Dade Chemistry Systems Inc., PO Box 6101, Newark, DE 19714). Each UTHC cartridge contains 20 tests of matched reagents.

The UTHC method is calibrated with a multianalyte liquid calibrator (Cat. No. DC40, Dade Chemistry Systems Inc., PO Box 6101, Newark, DE 19714) that contains eight drugs of abuse analytes in a human urine matrix. The cut-off for the UTHC method is 50 ng/ml (11-nor-$\Delta^9$-THC-9-carboxylic acid).

Positive and negative multianalyte liquid controls (Cat. No. DC43 and DC44, Dade Chemistry Systems Inc., PO Box 6101, Newark, DE 19714) have THC concentrations at approximately 25 and 75 ng/ml 11-nor-$\Delta^9$-THC-9-carboxylic acid, respectively.

### *Other materials*

The UTHC method on the aca® discrete clinical analyser was used in accordance with the manufacturers instructions (Dade Chemistry Systems Inc., PO Box 6101, Newark, DE 19714). Lyphochek® urine toxicology controls were obtained from Bio-Rad (ECS Division, 3726 E. Miraloma Ave., Anaheim, Ca., 92806). The ONLINE® THC calibrator was obtained from Roche Diagnostic Systems, Inc. (Branchburg, NJ 08876-1760).

### *Instrumentation*

The UTHC method is designed to run on Dimension® AR and XL clinical chemistry systems (Dade Chemistry Systems Inc., PO Box 6101, Newark, DE 19714).

### *Assay principle*

Each test is initiated by the addition of 90 µl of diluent and 115 µl of monoclonal antibody to the reaction cuvette. After a 60 s incubation period, 26 µl of sample and 59 µl of water are added to the reaction. After 78 s, 60 µl of microparticle reagent, with cannabinoid derivative bound to the particle surface, are added. Cannabinoids, if present in the sample, compete with the microparticle reagent for the antibody, thereby decreasing the rate of aggregation. Thus, the rate of aggregation is inversely proportional to the concentration of cannabinoids present in the sample. The rate of particle aggregation, as measured by absorbance changes at 510 and 700 nm, is determined over the next 143 s.

The absorbance change at the cut-off (50 ng/ml) calibrator is normalized during calibration to provide a reference numerical value arbitrarily set to 1000. These

0142-0453/97 $12·00 © 1997 Taylor & Francis Ltd units are referred to as 'normalized qual units'. Negative results are less than 1000 normalized qual units and positive results are greater than or equal to 1000 normalized qual units. The instrument automatically calculates and prints the results of the UTHC analysis in normalized qual units, as well as the interpretation of the result as positive or negative.

### Results

### Assay optimization

Antibody, particle reagent, diluent and sample volumes were determined using simultaneous variable co-optimization (RS/Discover by Bolt, Beranek and Newman, Inc.) and linear optimization techniques. Diluent volume had the most pronounced effect on the rate of particle aggregation, especially with drug-free samples. Sample size was the most significant factor to influence the shape of the calibration curve (figure 1). A 26 µl sample size was selected to provide optimal precision around the 50 ng/ml cut-off, to provide a reasonable calibration curve up to 100 ng/ml and to minimize false positive and false negative results with clinical samples.

### Reproducibility

For within-run and total reproducibility experiments, the method was calibrated on the first day of the study. Calibrator, quality controls and urine pools were then analysed in duplicate for 20 non-consecutive days without recalibration. The within-run and total coefficients of variation (% CV), expressed as normalized qual units, were calculated using analysis of variance (NCCLS protocol EP5-T2). The results, shown in table 1, indicate that within-run reproducibility is less than 0·6% and total reproducibility is less than 1·6% at all levels tested. Expressed in concentration units (ng/ml), the within-run and total reproducibility at the 50 ng/ml cut-off are 2·1% and 6·5%, respectively.

In addition, the within-run reproducibility of the method was further tested using drug-free urine samples spiked with 40 and 60 ng/ml 11-nor-$\Delta^9$-THC-9-carboxylic acid. These samples were tested with 20 replicates per level on two days across five analysers. All of the 400 results were classified correctly as positive or negative compared to the 50 ng/ml cut-off calibrator.

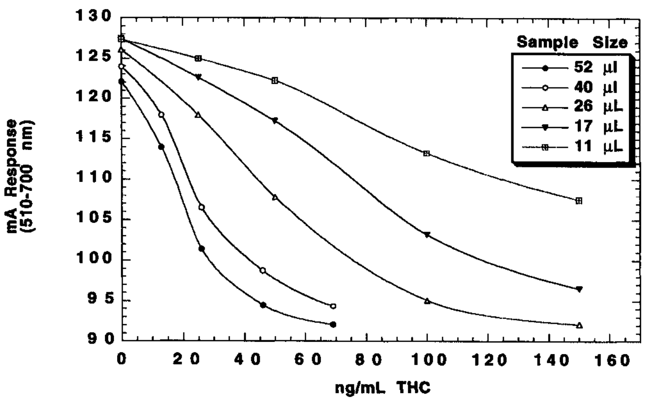

*Figure 1. Effect of sample size on shape of calibration curve.*

*Table 1. Reproducibility of the UTHC method using NCCLS protocol EP5-T2.*

| Material | ng/ml | Mean (Normalized qual units) | %CV (Normalized qual units) | |
|---|---|---|---|---|
| | | | Within-run | Total |
| Urine pool | 20 | 877 | 0·42 | 0·60 |
| ONLINE® THC calibrator | 25 | 897 | 0·37 | 1·0 |
| ONLINE® THC calibrator | 50 | 996 | 0·46 | 1·51 |
| Lyphocheck Screen control | 60 | 1091 | 0·52 | 0·90 |
| Urine pool | 86 | 1123 | 0·57 | 0·75 |
| ONLINE® THC calibrator | 100 | 1131 | 0·50 | 0·92 |
| Lyphocheck Screen control | 164 | 1133 | 0·41 | 0·46 |

(Lyphocheck is a registered trademark of BIO-RAD, ECS Division, 3726 E. Miraloma Avenue, Anaheim, CA 92806.)

Sample carry-over was evaluated by interleaving five replicate groups of drug-free urine samples and a sample containing 1886 ng/ml 11-nor-$\Delta^9$-THC-9-carboxylic acid. No detectable sample carry-over (<2 ng/ml) was observed in any of the drug-free samples.

### Stability

To determine the calibration interval for the UTHC method, the method was calibrated on day 1 with the 50 ng/ml cut-off calibrator and samples containing 25, 60 and 100 ng/ml 11-nor-$\Delta^9$-THC-9-carboxylic acid were tested (figure 2). The normalized qual units at each of the levels were converted to analyte concentration (ng/ml) using a logit equation and a linear regression analysis performed ($y$ = ng/ml THC, $x$ = day). The slopes of these regression equations were −0·007, 0·155, and

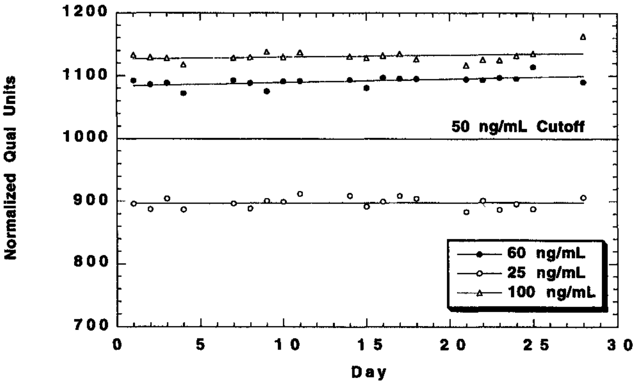

*Figure 2. Calibration stability: urine controls containing 25, 60 and 100 ng/ml 11-nor-$\Delta^9$-THC-9-carboxylic acid were processed using four replicates per level per day. Results are expressed as normalized qual units calculated using the day 1 calibration.*

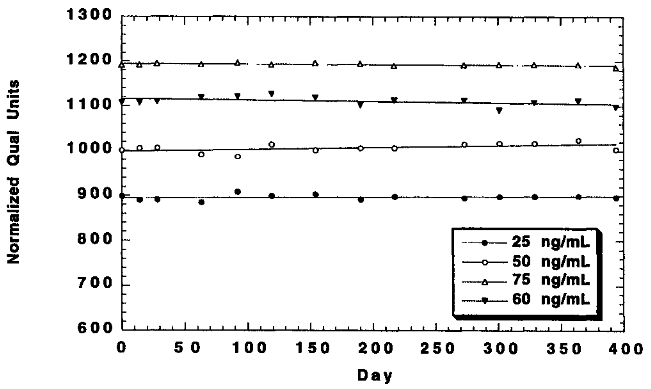

*Figure 3. Long term stability: the mean results of 10 replicate sets per level were obtained from reagents calibrated on day 1 of the study.*

0·147 ng/ml per day at the 25, 60 and 100 ng/ml 11-nor-$\Delta^9$-THC-9-carboxylic acid concentrations, respectively. From these regression slopes, the analyte concentrations had changed less than 10% after 30 days. Based on these results, and results from multiple reagent lots, calibration of the UTHC method was determined to be stable for at least 30 days.

The long-term reagent stability of the UTHC method was determined by testing urine controls containing 25, 50, 60 and 75 ng/ml 11-nor-$\Delta^9$-THC-9-carboxylic acid for 394 days. The means of the normalized qual units ($\mathcal{N} = 10$ on each test date) at each of these levels is shown in figure 3.

### *Specificity*

The specificity of the UTHC method to various cannabinoids and cannabinoid metabolites was determined by spiking each compound into drug-free human urine and determining the approximate quantity needed to give a response equivalent to that of the 50 ng/ml 11-nor-$\Delta^9$-THC-9-carboxylic acid cut-off calibrator (table 2).

To additionally characterize the method's specificity, 79 non-structurally related drugs were added to aliquots of pooled drug-free normal human urine at a concentration of 100 000 ng/ml and found to be negative in the UTHC method (table 3).

Potential interferences for the UTHC method were tested by adding various physiological substances and other compounds to a human urine pool containing 50 ng/ml 11-nor-$\Delta^9$-THC-9-carboxylic acid. All responses from these samples corresponded to concentrations between 45 and 55 ng/ml 11-nor-$\Delta^9$-THC-9-carboxylic acid (table 4).

### *Method comparison*

Four hundred and forty five urine specimens were tested for cannabinoids using the Dimension and aca analysers. The cannabinoid method on the aca analyser is based on EMIT reagents (EMIT is a registered trademark of Behring Diagnostics, Inc., San Jose, CA 95161-9103). For both systems, results greater than or equal to the

*Table 2. The concentration of cannabinoids required to produce a response equivalent to that of the 50 ng/ml cut-off calibrator*

| *Compound* | *Concentration equivalent to 50 ng/ml THC cut-off ng/ml* |
|---|---|
| 8-α-Hydroxy-$\Delta^9$-THC | 157 |
| 11-Hydroxy-$\Delta^9$-THC | 386 |
| 8-β-11-Dihydroxy-$\Delta^9$-THC | 344 |
| $\Delta^9$ THC | 242 |
| 11-Hydroxy-cannabinol | 2714 |
| Cannabinol | 1360 |
| 11-Nor-$\Delta^9$-THC-9-carboxylic acid | 50 |

50 ng/ml calibrator were considered positive. All samples that tested positive by either or both methods were confirmed by GC/MS. Results are shown in table 5. One sample was negative on the aca analyser and positive by both Dimension® and GC/MS (86 ng/ml). Three samples were positive on the aca® analyser and negative on Dimension®. These samples contained THC concentrations of 12, 15 and 17 ng/ml by GC/MS.

### Discussion

A qualitative screening method for the detection of cannabinoids (UTHC), along with a multianalyte liquid calibrator, and a positive and negative quality control, have been developed for the Dimension® AR and XL clinical chemistry systems. The UTHC method running in a random access mode delivers a result in 9 min. Studies (data not shown) where the UTHC method was interleaved with other general chemistry methods, demonstrated no interference.

The Dimension® cannabinoid method uses ONLINE® reagents and instrument specific method parameters that were optimized for method reproducibility and clinical performance using a single point 50 ng/ml calibrator. Previous reports have demonstrated that lowering the cannabinoid cut-off from 100 to 50 ng/ml can increase efficiency and sensitivity with only a minor decrease in specificity [2]. In addition, the US Department of Health and Human Service Guidelines for forensic urine drug testing now specify a 50 ng/ml cut-off.

Within-run and total reproducibility, determined using a 20 day NCCLS protocol (EP5-T2) were excellent for the UTHC method. While authors often compare reproducibility for drug of abuse methods in terms of absorbance units, it is more appropriate to transform absorbances to concentrations and to compare reproducibility in concentration units [3]. The normalized qual units as shown in table 1 were converted to concentration units. At the 50 ng/ml cut-off the within-run and total reproducibility were 2·1 and 6·5% respectively.

The calibration stability results presented in figure 2 clearly indicate that the method retains acceptable calibration for at least one month. Results from the long-

*Table 3. 100 000 ng/ml of these compounds were added to drug-free human urine and determined to give a negative result in the UTHC method.*

| | | |
|---|---|---|
| Acetaminophen | Ecgonine | Morphine |
| Acetylsalicylic acid | Ecgonine methyl ester | Naloxone |
| Aminopyrine | Ephedrine | Naltrexone |
| Amitriptyline | Epinephrine | Naproxen |
| Amobarbital | Erythromycin | Niacinamide |
| Amphetamine | Estriol | Norethindrone |
| Ampicillin | Fenoprofen | Oxazepam |
| Ascorbic acid | Furosemide | Penicillin G |
| Aspartame | Gentisic acid | Pentobarbital |
| Atropine | Glutethimide | Phencyclidine |
| Benzocaine | Guaiacol glycerol ether | Phenobarbital |
| Benzoylecgonine | Hydrochlorothiazide | Phenothiazine |
| Benzphetamine | 5-Hydroxyindole-3-acetic acid | Phenylbutazone |
| Butabarbital | | Phenylpropanolamine |
| Caffeine | 5-Hydroxyindole-2-carboxylic acid | Procaine |
| Calcium hypochlorite | | Promethazine |
| Chlordiazepoxide | Ibuprofen | Quinidine |
| Chloroquine | Imipramine | Quinine |
| Chlorpheniramine | Isoproterenol | Secobarbital |
| Chlorpromazine | Ketamine | Sulindac |
| Cocaine | Lidocaine | Tetracycline |
| Codeine | LSD | Tetrahydrozoline |
| Dextromethorphan | Melanin | Trifluoperazine |
| Dextropropoxyphene | Meperidine | Verapamil |
| Diazepam | Methadone | Zomepirac |
| Diphenhydramine | d-Methamphetamine | |
| Diphenylhydantoin | Methaqualone | |
| Dopamine | Methyprylon | |

*Table 4. These compounds were added at the concentrations shown to a human urine pool containing 50 ng/ml 11-nor-$\Delta^9$-THC-9-carboxylic acid and tested in the UTHC method.*

| | | | |
|---|---|---|---|
| Ammonia | 1·8 mg/l | Magnesium | 1·0 g/l |
| Ascorbic acid | 1·0 g/l | Methsuximide | 0·08 g/l |
| Amikacin | 0·2 g/l | NaOH | 0·1% |
| Bilirubin | 20 mg/l | Nicotine | 0·1 g/l |
| Boric acid | 0·5% | Osmolality (as NaCl) | 50 mOsmol/kg |
| Calcium | 1·5 g/l | | 1400 mOsmol/kg |
| Carbamazepine | 0·2 g/l | Oxalic acid | 1·0 g/l |
| Chloramphenicol | 0·3 g/l | Penicillin V | 0·8 g/l |
| Cimetidine | 0·01 g/l | Phenytoin | 0·1 g/l |
| Citric acid | 2·0 g/l | Phosphorus | 5·0 g/l |
| Creatinine | 8·0 g/l | Primidone | 0·1 g/l |
| Digoxin | 3·0 μg/l | Protein | 120 g/l |
| Ethanol | 3·5 g/l | Salicylic acid | 0·1 g/l |
| Ethosuximide | 0·3 g/l | Sodium azide | 0·5% |
| Gentamicin | 0·2 g/l | Sodium fluoride | 0·01 g/l |
| Glucose | 25 g/l | Theophylline | 0·1 g/l |
| HCl | 0·1 N | Thimerosal | 0·1% |
| Haemoglobin | 5·0 g/l | Thymol | 2·0 g/l |
| Heparin (porcine) | 8000 U/l | Urea nitrogen | 17 g/l |
| Ketones (acetone) | 0·4 g/l | Uric acid | 0·15 g/l |
| Lithium | 0·04 g/l | Valproic acid | 0·5 g/l |

term reagent stability study, shown in figure 3, demonstrate that the flex reagents are stable for at least one year after being manufactured.

The specificity of the UTHC method to various cannabinoids and cannabinoid metabolites is shown in table 2. The single most abundant marijuana metabolite found in urine after smoking or ingestion is 11-nor-$\Delta^9$-THC-9-carboxylic acid [4]. Therefore, a monoclonal antibody having good reactivity to this metabolite was utilized in the method. As 11-nor-$\Delta^9$-THC-9-carboxylic acid is used to calibrate this method, samples containing greater than 50 ng/ml 11-nor-$\Delta^9$-THC-9-carboxylic acid, or other metabolites having reactivity equivalent to a 50 ng/ml calibrator, will produce a positive result in the method.

*Table 5. Cannabinoid method comparison between the Dimension® and aca® analysers.*

| | aca® Positive | aca® Negative |
|---|---|---|
| Dimension® positive | 85 | 1 |
| Dimension® negative | 3 | 356 |

The clinical performance of the Dimension® UTHC method was demonstrated by comparison to an EMIT based method on the aca® analyser using 445 samples (table 3). A 99·1% agreement between these methods was observed. Three samples, positive on the aca® and negative on Dimension®, with immunochemical reactivity near the cut-off of 50 ng/ml, contained concentrations of 12–17 ng/ml by GC/MS. For the population of samples evaluated in this study, aca® (EMIT® reagents) and Dimension® (ONLINE® reagents) detected 99% and 97%, respectively, of samples confirmed positive by GC/MS. All positive cannabinoid results were confirmed as positive by GC/MS utilizing a cut-off of 10 ng/ml. These differences in cannabinoid detection rates are typical. RIA (Roche Abuscreen) and ONLINE® reagents (on a Hitachi 717 analyser) detected 99% of a population of 353 GC/MS confirmed positive samples, whereas EMIT® II reagents (on a Hitachi 717 analyser) detected 88% and TDx® (TDx is a registered trademark of Abbott Laboratories) detected 95% of these same samples [5]. In another study of 100 EMIT® positive THC samples, the Triage® Panel for Drugs of Abuse (Triage is a registered trademark of Biosite Diagnostics, Inc.) detected 93% [6]. Using the Hitachi 717 analyser with EMIT® II, CEDIA® (CEDIA is a registered trademark of Microgenics Corporation) and ONLINE® reagents, and a subset of 188 GC/MS confirmed positive samples from 18 800 total screened samples, RIA (Immunalysis kits), EMIT® II, CEDIA®, ONLINE® and TDx® detected 100, 86, 89, 89 and 87%, respectively, of the positives samples [7]. Variation of detection rates between immunoassay systems is probably due to differences among individual drug users' metabolism along with differences in antibody specificity [8,9].

In conclusion, the cannabinoid method developed for use on the Dimension® AR and XL chemistry systems provides a precise, accurate and rapid screening approach for measuring cannabinoids on these general chemistry analysers. Since the Dimension® is used for the measurement of many other analytes in a true random access mode, the benefits of workstation consolidation may be realized.

### References

1. Knight, D., Singer, R., White, J. M. and Fraser, C. G., *Clinical Chemistry*, **34** (1988), 1899–1903
2. Huestis, M. A., Mitchell, J. M. and Cone, E. J., *Clinical Chemistry*, **5** (1994), 729–773.
3. Smith, F. P., *Clinical Chemistry*, **40** (1994), 1782–1783.
4. King, D. L., Martel, P. A. and O'Donnell, C. M., *Clinics in Laboratory Medicine*, **7** (1987), 641–653.
5. Armbruster, D. A., Schwarzhoff, R. H., Hubster, E. C. and Liserio, M. K., *Clinical Chemistry*, **39** (1993), 2137–2146.
6. Wu, A. H. B., Wong, S. S., Johnson, K. G., Callies, J., Shu, D. X., Dunn, W. E. and Wong, S. H. Y., *Journal of Analytical Toxicology*, **17** (1993), 241–245.
7. Armbruster, D. A., Hubster, E. C., Kaufman, M. S. and Ramon, M. K., *Clinical Chemistry*, **41** (1995), 92–98.
8. Colbert, D. L., *British Journal of Biomedical Sciences*, **51** (1994), 136–146.
9. Wells, D. J. and Barnhill, M. T., *Clinical Chemistry*, **35** (1989), 2241–2243.

